# Multiple Breeds and Countries’ Predictions of Mineral Contents in Milk from Milk Mid-Infrared Spectrometry

**DOI:** 10.3390/foods10092235

**Published:** 2021-09-21

**Authors:** Octave S. Christophe, Clément Grelet, Carlo Bertozzi, Didier Veselko, Christophe Lecomte, Peter Höeckels, Andreas Werner, Franz-Josef Auer, Nicolas Gengler, Frédéric Dehareng, Hélène Soyeurt

**Affiliations:** 1 Walloon Agricultural Research Center (CRA-W), 24 Chaussée de Namur, 5030 Gembloux, Belgium; o.christophe@cra.wallonie.be (O.S.C.); c.grelet@cra.wallonie.be (C.G.); 2 Elevéo Asbl, AWE Group, 4, Rue des Champs Elysées, 5590 Ciney, Belgium; cbertozzi@awenet.be; 3 Comité du Lait de Battice Route de Herve 104, 4651 Battice, Belgium; didier.veselko@comitedulait.be; 4 France Conseil Elevage, Maison du Lait, 42 Rue de Chateaudun, 75009 Paris, France; christophe.lecomte@france-conseil-elevage.fr; 5 Landeskontrollverband Nordrhein-Westfalen e.V., Bischofstraße 85, 47809 Krefeld, Germany; hoeckels@lkv-nrw.de; 6 LKV Baden Württemberg, Heinrich-Baumann Str. 1-3, 70190 Stuttgart, Germany; awerner@lkvbw.de; 7 LKV Austria Gemeinnützige GmbH, Dresdnerstr. 89/B1/18, 1200 Wien, Austria; Franz-Josef.Auer@lkv-austria.at; 8 Gembloux Agro-Bio Tech, TERRA Teaching and Research Centre, University of Liège, 5030 Gembloux, Belgium; nicolas.gengler@uliege.be (N.G.); hsoyeurt@uliege.be (H.S.)

**Keywords:** milk, mid-infrared, minerals

## Abstract

Measuring the mineral composition of milk is of major interest in the dairy sector. This study aims to develop and validate robust multi-breed and multi-country models predicting the major minerals through milk mid-infrared spectrometry using partial least square regressions. A total of 1281 samples coming from five countries were analyzed to obtain spectra and in ICP-AES to measure the mineral reference contents. Models were built from records coming from four countries (*n* = 1181) and validated using records from the fifth country, Austria (*n* = 100). The importance of including local samples was tested by integrating 30 Austrian samples in the model while validating with the remaining 70 samples. The best performances were achieved using this second set of models, confirming the need to cover the spectral variability of a country before making a prediction. Validation root mean square errors were 54.56, 63.60, 7.30, 59.87, and 152.89 mg/kg for Na, Ca, Mg, P, and K, respectively. The built models were applied on the Walloon milk recording large-scale spectral database, including 3,510,077. The large-scale predictions on this dairy herd improvement database provide new insight regarding the minerals’ variability in the population, as well as the effect of parity, stage of lactation, breeds, and seasons.

## 1. Introduction

Measuring the mineral composition of milk is of major interest in the dairy sector as they play an important role for human and animal health, and also due to milk’s technological properties and the environmental impact of dairy farming. The main minerals in bovine milk are calcium (Ca, around 1200 mg/kg), potassium (K, around 1500 mg/kg), sodium (Na, around 350 mg/kg), magnesium (Mg, around 90 mg/kg), and phosphorus (P, around 950 mg/kg) [1]. Among others, calcium in the human diet affects the rigidity of skeletal and neuromuscular function, enzyme-mediated processes, and blood clotting [2], as well as arterial hypertension, colon cancer [3,4], and regulation of body weight and body fat [4,5]. Potassium plays a fundamental role in the maintenance of homoeostasis and participates in the transmission of nervous impulses, muscle contraction, and regulation of blood pressure [6]. An increase of dietary potassium, coupled with a reduction of dietary Na, limits the risk of hypertension [7]. Uribarri and Calvo (2014) [8] claimed that high dietary P intake, although considered an essential nutrient, is a risk factor for bone and cardiovascular diseases.

Besides health aspects, Ca and P are essential components of casein micelles [9,10] and are consequently directly involved in the milk coagulation process. In particular, they have a strong influence on the ability of milk to coagulate and on the final consistency of the coagulum [11].

The mineral profile of milk can also be considered as a biomarker for cow management, especially regarding udder health. Both Na and K in milk are affected by ruptured mammary epithelia and alteration of blood–milk barriers induced by mastitis [12,13,14]. Basin et al. [15] also observed that a higher risk of clinical mastitis was genetically strongly associated with greater variation over the lactation of the contents in milk of Na and Ca predicted from milk mid-infrared (MIR) spectrometry. K and Na were also mentioned to be associated with metabolic disorders, such as mastitis, alkalosis, and acidosis [13].

Finally, the P content in milk could be used in order to better manage the environmental impact of dairy herds. Indeed, through effluents, dairy farms are participating in the release of phosphate in surface and ground water resources [16], promoting eutrophication of aquatic systems. This results in the bloom of aquatic plants, growth of algae, and depletion of dissolved oxygen [17]. In Europe, the EU Nitrates Directive (European Council, 1991) is the core regulation to limit nutrient loss from farming systems. To comply, countries may apply drastic plans for the dairy sector, such as the phosphate regulation plan in the Netherlands leading to a reduction by 11% of the number of dairy cows [18]. Therefore, measuring milk P would allow better insight on the needs of cow P and P efficiency in order to reduce the environmental impact.

Consequently, there is a need to assess the mineral profile in milk through cost-effective and large-scale methods, such as milk MIR spectrometry. From 2009 and the first study conducted by Soyeurt et al. [19], numerous studies were carried out to develop MIR models predicting milk minerals [20,21,22,23,24,25,26]. For wide, large-scale use by dairy herd improvement (DHI) centers and dairy industries, such models need to be robust to provide accurate and reproducible predictions in various conditions. To achieve this objective, large variability in the calibration dataset both regarding the reference values and the spectral data is essential [27,28]. For this reason, the first objective and the novelty of this work is to develop MIR models predicting the mineral content of milk from a large and variable dataset, including records coming from several countries, breeds, seasons, diets, and MIR spectrometers. No models presented in the literature [20,21,22,23,24,25,26] exhibit a multi-country approach, and the models presented could allow a wide range of utilization for the farmer and could be used for all the countries present in the calibration set. The second objective was to test the models on a country-independent validation dataset and to evaluate the impact of including local samples in the calibration dataset. The third objective was to study the different correlations between minerals and other traits to confirm the potential interest of those predictions as animal health or milk technological indicators. Finally, the built models were applied on the millions of spectra present in the Walloon DHI database managed by the Walloon Breeding Association in order to get more insight on the behavior of those predicted mineral contents in milk from a large-scale population of cows.

## 2. Materials and Methods

### 2.1. Data

From 2005 to 2020, approximately 3000 milk samples were collected from individual cows in five different countries (Belgium, France, Germany, Luxembourg, and Austria). Those samples were collected by local DHI centers with the objective to maximize the variability, by sampling local specificities, diets, breeds, and management systems. Based on this methodology, the collected samples represented 13 dairy breeds: Abondance (ABO), Aubrac (AUB), dual-purpose Belgian Blue (BBL), Brown Swiss (BSW), Fleckvieh (FLE), Holstein-Friesian (HOL), Jersey (JER), Meuse-Rhine-Yssel-type Red and White (MRY), Montbéliarde (MON), Normande (NMD), Salers (SAL), Simmental (SIM), and Tarentaise (TAR).

As the measurement of the mineral content by chemical analysis is costly and time-consuming, we try to limit the number of analysis. To gather robustness to the model while limiting the chemical analysis, only half of those samples were selected based on their spectral variability. Starting from the model developed by Soyeurt et al. [19], new samples were added iteratively, by picking the most spectrally different samples. The sample selection was based on the standardized Mahalanobis distance, also called the Global H distance (GH), and the neighboring H distance (NH) [27]. This led to a dataset of 1281 selected samples, with 502 samples from Belgium and Luxembourg, 388 samples from France, 291 samples from Germany, and 100 samples from Austria.

All milk samples were collected following the guidelines edited by the International Committee for Animal Recording and with the use of a preservative to ensure the stability of milk physico-chemical properties before analysis. To be representative of the real routine methods, the samples coming from different countries were preserved using the antimicrobial and concentration classically used within each local milk recording. In the majority of cases, bronopol was used with a concentration of 0.03%, in some places with an addition of Kathon${}^{\mathrm{TM}}$ CG (mix of Methylchloroisothiazolinone (CMIT) and Methylisothiazolinone (MIT); Dow Chemical Company, Midland, MI, USA) at 0.003%. For 136 samples, sodium azide tabs were used as antimicrobial. As the use of sodium azide distorted the Na analysis because ICP-AES took into account the sodium concentration contained in the antimicrobial product, we chose to remove the associated 136 Na reference values. MIR spectra were generated by spectrometers from the analysis of fresh milk samples by the local milk laboratories. The different spectrometers used were FT6000, FT+ and FT7 (Foss, Hillerød, Denmark), FTS (Bentley, Chaska, MN, USA), and Standard Lactoscope FT-MIR automatic (PerkinElmer, Waltham, MA, USA). To harmonize the spectral information, the spectral data were standardized into a common format using the piece-wise standardized method (PDS) following the OptiMIR project protocol [29]. Since this standardization method was developed in 2011, only the spectra recorded after this date were standardized (i.e., the last 986 out of 1281). However, the remaining raw spectra (not standardized) were all generated by Foss spectrometers based on which the common format was established.

### 2.2. Reference Analysis

The inductively coupled plasma-atomic emission spectroscopy (ICP-AES) (Horiba Jobin Yvon GmbH, Bensheim, Germany) was utilised as the reference method for the determination of the mineral content in milk. The dispersive system was a Czerny–Turner monochromator with a focal distance of 1 m. The wavelength pathways ranged from 120 to 800 nm.

Humidification of argon was used, as well as a peristaltic pump. The integration parameters were strictly the same as described by Soyeurt et al. [19]: nebuliser gas flow = 0.75 min${}^{-1}$; pressure of nebuliser = 3 bar; Rf power = 1100 W; fixed time of rising = 60 s; fast-speed rinsing pumps; time of transfer = 15 s; time of stabilisation = 45 s; fast speed of transfer pump; synchronisation time = 0 s; and normal speed of pumps = 20 m/s. The wavelength for the determination of Na, Ca, Mg, P, and K were 590, 318, 279, 178, and 766 nm, respectively.

As described by Soyeurt et al. [19], the use of a previous mineralisation stage of the sample was ignored in order to avoid any mistakes from the losing sample. A dilution method described by Murcia et al. [30] was chosen thanks to the conclusion by Soyeurt et al. [19]. Direct quantification was performed using a dilution factor milk sample of 100. To improve the repeatability of the measurement, an addition of 1 mL surfactant Triton X-100 (Polyethylene glycol tert-octylphenyl ether and Sigma-Aldrich Birnem, Belgium) concentrated at 1 M was performed.

Therefore, the ICP-AES technique was carried out without mineralization to perform a direct quantification on diluted milk samples (1:100).

In addition, the removal of Na values due to the use of sodium azide tabs, 30, 0, 43, and 35 samples were rejected for Ca, Mg, P, and K, respectively, principally because of low repeatability induced by poor sample preservation or analytical issues.

### 2.3. Modelling

The pretreatments consisted of applying a first derivative with a gap of five wavenumbers and a normalization of variables (autoscale). Then, to limit the presence of noisy areas, the spectral area selected was like the region selected by Grelet et al. [27]: 968.1 to 1577.5 cm${}^{-1}$, 1731.8 to 1762.6 cm${}^{-1}$, 1781.9 to 1808.9 cm${}^{-1}$, and 2831.0 to 2966.0 cm${}^{-1}$. This represents the 212 first-derivative spectral points.

Calibration and cross-validation were performed on dataset-merging records coming from Belgium, Luxembourg, France, and Germany (maximum of 1181 samples). Calibrations were performed with partial least square (PLS) regression. Cross-validations were performed using 10 folds randomly constituted. During those cross-validations, samples with an absolute residual higher than 2.5 times the standard deviation of the global residuals were recognized as outliers and excluded from the models [31,32]. The number of latent variables (LV) were selected visually through the point of break of root mean square error of cross-validation (RMSEcv) slope. In other words, at this break point, adding a new LV did not substantially reduce the RMSEcv. However, a maximum of 16 LV was set to avoid overfitting [33].

In order to have a fully independent validation, the records coming from Austria were used to validate the developed models, as those samples came from a completely different environment; in particular, some of them were collected in the area of the Alps mountains with associated typical diets. All models were evaluated through the calculation of RMSE, R², and the ratio of performance to deviation (RPD), after both cross-validation and validation processes. As the RPD aims to combine the variability of the model (i.e., the SD of the calibration dataset) and the accuracy of predictions (i.e., the RMSE in the cross-validation and validation steps), it was calculated using the SD of the calibration dataset and the RMSE in the cross-validation and validation steps.

Then, in order to check the robustness of the forecasts (i.e., to assess the relevancy of having data coming from a country where we want to make a prediction), a second calibration set was created by adding, to the first training set, 30 randomly selected Austrian samples. The modeling process was exactly the same and the validation was performed on the 70 remaining Austrian samples to validate the model with four countries (Belgium, Luxemburg, France, and Germany) and the other one with the Austrian data included. Finally, all the samples were merged, and a third calibration dataset was created by randomly selecting 80% of randomly selected samples from the entire dataset (1024 of 1281 samples). Models were then validated with the remaining 20% of records.

Another metric was used to evaluate our models. The concordance correlations coefficient (CCC) allowed us to underline potential slope or bias problems in the development of the mineral models. The *CCC* was calculated through the Equation (1).

(1) $$CCC=\frac{2\rho \sigma_{x}\sigma_{y}}{\sigma_{x}^{2}+\sigma_{y}^{2}{\left(\mu_{x}+\mu_{y}\right)}^{2}},$$

where *x* is referring to the reference, and *y* to the predicted values; μ are the means, σ is the covariance, and ρ is the correlation coefficient between the reference and the predicted.

The different percentage of model improvement was calculated as stated in Equation (2).

(2) $$\%=\frac{\left(X-X_{aust}\right)}{X_{aust}},$$

where *X* can be the RMSECV, RMSEP, or RPD of model without any Austrian data included and $X_{aust}$ is with 30 Austrians included.

All computations were carried out with the PLS toolbox v. 4.11 (Eigenvector Research, Inc.,Wenatchee, WA, USA), Winisi software (version 4.6; Foss, Hillerød, Denmark), and scripts developed in Matlab v7.5.0 (The Mathworks, Inc., Natick, MA, USA).

### 2.4. Relationships with Traits Related to Animal Health and Milk Technological Properties

In order to assess the relevancy of considering major minerals as health indicators or milk technological indicators, the reference mineral contents were correlated with the MIR-based predictions known to be related to those issues. These traits are listed in Table 1 with their corresponding RPD. Moreover, in order to compare the relevancy of mineral predictions compared to the reference values, the mineral predictions were also correlated with the traits mentioned in Table 1.

The Pearson correlations between minerals and traits were calculated using the Equation (3). Two sorts of correlations are calculated in this study. The correlations correspond to the link between the reference and predicted minerals and predicted traits.

(3) $$\rho_{x,y}=\frac{\sigma_{\left(x,y\right)}}{SD_{x}SD_{y}},$$

where *x* is referring to mineral traits (reference or predicted) and *y* to other traits; ρ are the correlations, σ is the covariance between *x* and *y*, and SD is the standard deviation.

### 2.5. Large-Scale Phenotyping

The developed MIR predictive models were finally applied to the DHI spectral database related to the southern part of Belgium and managed by the Walloon Breeding Association (Awé, Ciney, Belgium). This database included 3,510,077 spectra from 235,355 different cows and 1344 herds recorded from 1 January 2012 to 4 March 2020. Records had days in milk (DIM) ranging from 5 to 365 days, and a parity up to 18 and concerning 16 different cows breeds. This large-scale prediction allowed us to obtain information about the evolution of milk mineral prediction following the stage of lactation, month of tests, and parity. These effects were assessed by computing descriptive statistics.

## 3. Results and Discussion

### 3.1. Reference Dataset

The descriptive statistics of the entire dataset are mentioned in Table 2. The various numbers of samples per mineral is related to the erroneous ICP-AES analysis, as mentioned previously. The main purpose of this study was to establish robust models who can be applied on a large application scale. Compared to the current study, the models published in the literature [19,23] had a different standard deviation and mean. Indeed, these metrics vary according to the sampling.

The models presented by Toffanin et al. [20] exhibited relatively few differences in mean concentration for Ca and P (0.84% and −5.84%) with our models. A higher average was especially found with the mineral models presented by Visentin et al. [23] (12.45%, 17.42%, 29.60%, 1.93%, −0.86% for Na, Ca, Mg, P and K, respectively). The sampling was carried out in the South Tyrol region where cattle are mainly used for cheese production.

Another difference is clearly exposed by Zaalberg et al. [26], and a mean difference is observed for Danish Holstein with −4.09%, 5.07%, 8.08%, −29.32%, and −5.49% and for Danish Jersey with 12.35%, 23.71%, 17.91%, −13.61%, and −14.49% for Na, Ca, Mg, P, and K, respectively, in comparison to our data.

### 3.2. Creation of Models Predicting Mineral Content in Milk

The prediction performances of both the different sets of predictive PLS models to determine the major mineral contents in milk are listed in Table 3. Maximum 1181 and 1211 samples were used to design both sets of calibration models. In order to ensure the comparison of results between the two sets of models, the number of LV used for the second set of models was the same as the ones fixed for the first set. The $\Delta_{outlier}$ has shown that the T test is mandatory in order to obtain unfalse models. Indeed, the T test decreased RMSE, especially for Ca and P. This decrease was probably due to a bad sampling or a precipitate issue coming from a long freezing time. The validation RPD value allowed us to assess the accuracy and robustness of each prediction equation. Both models had relatively similar cross-validation performances, but in cross-validation, RPD decreased from −1% to −13% when 30 Austrian samples were added in the training set. However, the validation RPD increased from 6 to 39%, except for Na, where RPD decreased by −16%. As stated in [27], it validates that inclusion of variability in the dataset can decrease the apparent accuracy (i.e., in cross-validation) but improve the robustness and real performances of the models in validation. It consequently seems important to cover the variability of new samples to predict in order to limit extrapolation and ensure better predictions in routine. Indeed, when the inclusion of Austrian dataset is added, the RMSEP decreases by 1.1%, 12.8%, 11.1%, 36.7%, and 18.7% for Na, Ca, Mg, P, and K, respectively.

The main purpose of using different sets of calibration was not to evaluate the model’s performance, but more to compare the data influence. Indeed, the data exposed a completely different distribution for both models. Within this study, the main purpose was to establish robust models, which is not classically the main criteria from a statistical point of view. In this specific case, a large coverage of spectral variability which implies that models can be applied in a large panel of cases was the priority. This sought-after robustness allows the model to be used on a larger scale for smart farming. The standard deviation performed with all the samples analysed from ICP-AES exhibited higher SD (Table 2) compared to the models exhibited in Table 3. When some values are too different from each other, the removal of these samples (considered as outliers) helps to minimize prediction errors.

The residual analysis performed during the calibration process made this job. The models were built in several sample collection phases, where the samples were selected if the predicted value from the previous model was of interest to improve the robustness of the newly created model. However, this data collection method is known to increase the prediction errors of a model [27].

The opposite results in cross-validation and validation show the relevancy of performing a fully independent validation. The RMSEP of validation mentioned in Table 3 confirms the importance of searching for variability to obtain the most robust model, as RMSEP was improved when only 30 Austrian samples were added in the training set. For cattle coming from a totally different environment with different feed rations, different climatic conditions, and different breeds, the milk composition could radically change. For Na, Ca, and Mg, a slight difference was found between both external validations (Table 3), which explains the model being robust enough to predict the Austrian samples well.

For the P model including Austrian samples in the calibration dataset, a large decrease of the RMSEP of validation was observed, confirming a better performance of the predicted model (i.e., Higher RPD). The relatively high errors of prediction (for both external validation) for the K model revealed the incapacity of the model to accurately predict the Austrian samples.

When 80% of samples in the calibration model were randomly chosen, the RMSEP for predicting K was 106.7 mg/kg. This prediction error seems more consistent (than the validation with only Austrian samples) and confirms the differences from K content in the Austrian milk sample. These results may be explained by the difficulty to predict the K content in milk.

Even with a robust model including samples from four different countries, the external validation exhibited some deficiencies to validate Austrian samples for P and K. A decrease for RMSEP was revealed when 30 samples coming from the Austrian validation set was included in the calibration set. The results obtained in this study highlight the need for data collaboration and the interest of including local variability to obtain the most robust models. The best way to collaborate is to have a common database and standardized spectra allowing to taking into account the machine-dependent errors [29].

Compared to the current study, the models published in the literature [15,19] exhibited similar model performance (RPD) but exhibited higher RMSE. Indeed, the RMSECV obtained in the current study were globally lower when compared to a previous study by −30%, −77%, −60%, +20%, and −49% [19] and −40%, −124%, −79%, −34%, and −31% [23] for Na, Ca, Mg, P, and K, respectively. However, it is important to note that the data exposed a completely different distribution in the other studies. Indeed, the results highlighted by the literature [19,23] present a higher mean, and consequently, a greater standard error of prediction. Consequently, it is more relevant to compare the results using the RPD, which is relative to RMSE and SD.

Another approach will be to compare with the *CCC* metric, but Soyeurt et al. [19] and Visentin et al. [23] did not evaluate their model with this parameter. When the *CCC* was applied to the cross-validations of our three models, the performance was approximately the same, except for Na coming from the first models (8.6%). The *CCC* informed us that our models were not subject to bias or slope issues.

Based on the RPD values [36], we can conclude that all models created for Ca and P have the potential for approximate quantitative predictions and screening, whereas the screening for Na, Mg, and K seems to be impossible due to the low RPD values of their models. However, the accuracy of those models was enough to discriminate different groups of cows producing high or low contents of minerals in their milk. The poor RPD performance of the model is related, so that the infrared does not directly detect minerals in milk. Indeed, since infrared measures the vibration of binding molecules, the infrared measurement cannot allow a direct detection of possible ions in solutions. The models presented are therefore indirect models.

### 3.3. Correlation between Mineral Traits and Traits Related to Animal Health and Milk Technological Properties

The Pearson correlations were estimated to achieve two objectives. The first one was to compare the reference minerals dataset with the MIR predicted traits, and the second one was to compare the predicted mineral dataset with the MIR predicted traits (Table 4). Since the dataset created to develop the different models was not representative of the population as the selection of samples was not randomly conducted, the correlations given in this study cannot be generalized to the population. However, it was interesting to observe whether the relationships between traits stayed similar when using reference or predicted mineral contents even if the prediction accuracy was not high. Moreover, this allows to confirm the relationships with other predicted traits known to be related to animal health or the milk technological properties.

When comparing the correlation obtained between reference or predicted mineral traits and other MIR predicted traits mentioned in Table 4, the value of correlations had the same trend, but not the same amplitude. Indeed, the correlations between the predicted mineral data generally result in a higher correlation than those using the reference values. This correlations amplification could be mainly explained by the common part of the spectral information shared by all the predicted traits. In order not to exaggerate the reality, the results and discussion will focus on the correlation values between the measured minerals and the predicted features.

Pearson’s correlation between the reference mineral contents of milk ranged from −0.27 to 0.55. Moderate relationships between Mg and P, Ca, and Mg, as well as Ca and P were found with a correlation value of 0.55, 0.54, and 0.49, respectively. Weak negative relationships were found for Na and K, Ca and K, as well as Mg and K with −0.27, −0.15, and −0.14, respectively. It is interesting to notice that the correlation between mineral contents predicted from the calibration set exposed the same trend of relationship (Table 4).

The caseins were part of the phosphoproteins with 0.85% of the phosphorous as phosphates. Unlike whey proteins, which do not contain any phosphorous, the phosphates are responsible for the ability to bind a large amount of calcium cations. This type of phosphate coming from the esterification of the hydroxyl group of serine was called organic phosphate [37]. The presence of calcium ions associated with casein led to the formation of casein micelles. This calcium ionic interaction is strongly linked to protein concentration [37]. The analysed minerals were initially found as Calcium or Magnesium Phosphate. As there were counterions (ionic interaction), a strong correlation between these minerals exist (Ca and P, Mg and P; Table 4). Magnesium is also involved in many enzymatic reactions. For example, in milk, Mg is involved with alkaline phosphatase, which has a role of dephosphorylating agents [38].

These phosphorous salts are mainly found inside large colloidal particles of casein under the form of colloidal (nano-clusters) phosphate. Indeed, the phosphorous is distributed within 38.5% of colloidal inorganic phosphate, 33% of inorganic salt soluble in milk, 20% of organic phosphate (casein phosphate), 7% as organic esters, and 1.5% of phosphorous lipids [39]. The percentage of the salt included in the micelle was around 6% (*w*/*w*) [37,40], explaining the link between pProteins and Mg and P found in Table 4. In order to better explain this phenomenon, the method and models developed by Franzoi et al. [24,40] could be used to calculate the correlation between casein and the micellar mineral.

In the aqueous phase of the milk, the bivalent cations could be favorably chelated to citrate to form undissociated complexes. The positive correlation between pCitrate and Ca and Mg (r = 0.43, 0.48, respectively) could explain the possible chelation.

Increased sodium in milk is a sign of udder infection in cows [41]. Therefore, the sodium comes from a blood transfer with the milk. To maintain a constant osmotic pressure, the synthesis of lactose is reduced [39,42]. The Na content was negatively correlated with pLactose (Table 4) confirming this transfer. It has been demonstrated that NAGase is a mastitis marker [43,44]. In this study, a positive correlation between Na and pNAGase and pLDH was found (Table 4) leading to a possible confirmation of the link between sodium and mastitic infection. Moreover, a clear positive correlation was found with pLactoferrin (r = 0.36 to 0.62), which is also known as a mastitis indicator [34,45].

High Na and low K content in milk were found to be a mastitis indicator [46]. This is in line with the negative correlation observed between these two minerals. Summer et al. [47] revealed the links between Na, K, and somatic cells confirming that low potassium content is an indicator of clinical mastitis [47]. When milk exhibits a high somatic cell content (>400,000), the Na concentration tends to increase, while the K content tends to drop.

The milk coagulation ability (k20, a30, and Fresh ILCY) was mainly correlated with Ca, Mg, and P (Table 4) and exposed the importance of mineral composition to cheese manufacturing [48]. Sanchez et al. [49] asserted a moderate genetic correlation between Ca, Mg, and P with fresh curd yield and coagulation traits. No genetic correlation was found with Na and K. These findings follow the same correlation as our study.

### 3.4. Descriptive Statistics Applied on the Walloon Database

Computations were carried out with scripts developed in R studio 1.2.5033 (RStudio, Inc., Boston, MA, USA) using R statistical software 3.6.2 (R development Core Team 2019). The descriptive statistics derived from the large-scale prediction are shown on the Table 5.

### 3.5. Month Influence on Milk-Predicted Mineral Composition

**Figure 1:**
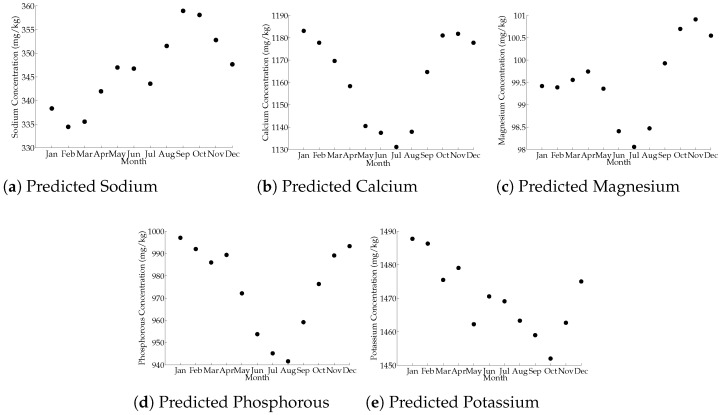
Evolution of predicted minerals in the function of the month when models are applied on a DHI milk spectral database. With a number of observations per month of: January (*n* = 330,321); February (*n* = 318,414); March (*n*= 318,433); April (*n* = 310,021); May (*n* = 314,933); June (*n* = 302,846); July (*n* = 139,963); August (*n*= 293,711); September (*n* = 290,688); October (*n* = 291,484); November (*n* = 298,209); December (*n* = 301,054). (**a**) Predicted Sodium (**b**) Predicted Calcium (**c**) Predicted Magnesium (**d**) Predicted Phosphorous (**e**) Predicted Potassium.

The descriptive statistics derived from large-scale prediction from the Walloon DHI database are shown in Table 5. By monthly averaging those results, the annual evolution of concentration of the five main predicted minerals in milk were computed and shown in Figure 1. There were three types of predicted mineral annual tendencies: (1) sodium (2) calcium, magnesium, and phosphorus, and (3) potassium. Predicted sodium increased from February to September, with a slight decrease between May and July. During the year, the lowest amount of pNa was around February, and the maximum amount around September. A local minimum was observed in July.

The second tendency exposed an annual behavior with a characteristic minimum during the summer and a significant increase between August and September. Although the concentration of pMg remained mostly stable between October and January, a sharp decrease was observed between December and January. For pCa, a similar plateau was observed between October and December.

The last predicted mineral tendency (K) experienced a sharp decline for a long period of the year between January and October. During this long period of decline, two local minima were observed in March and May, with the strongest decline occurring between April and May.

For pCa and pP, the concentration followed the same trend as the fat concentrations depending on the month, as shown by Zhang et al. [50], while pMg was more similar to the proteins aspect. Bittante et al. [51] has also shown that the proteins and the fat in milk decrease during the summer. Thus, the predicted minerals Ca, Mg, and P followed an identical pathway.

Diet is a key factor in explaining this behavior: throughout the year, the diet can change radically. In the studied region, during the summer, the livestock were mainly on pastures, and little supplementary feed (concentrate, silage, etc.) was given, resulting in a lower proportion of fat and protein. Grazing is also susceptible to modify the cation/anion balance of the diet, which affects the body mineral balance [52]. Another important change during the summer is the increase in temperature. This heat stress had a negative impact on the cow’s well-being, leading to underfeeding and therefore a modification of milk composition [50].

In many countries, farmers use group calving at the same time of the year. This induces cows to have the same stage of lactation at the same time, which may explain some of the differences in mineral content throughout the year.

### 3.6. Effect of Lactation on Milk-Predicted Minerals

**Figure 2:**
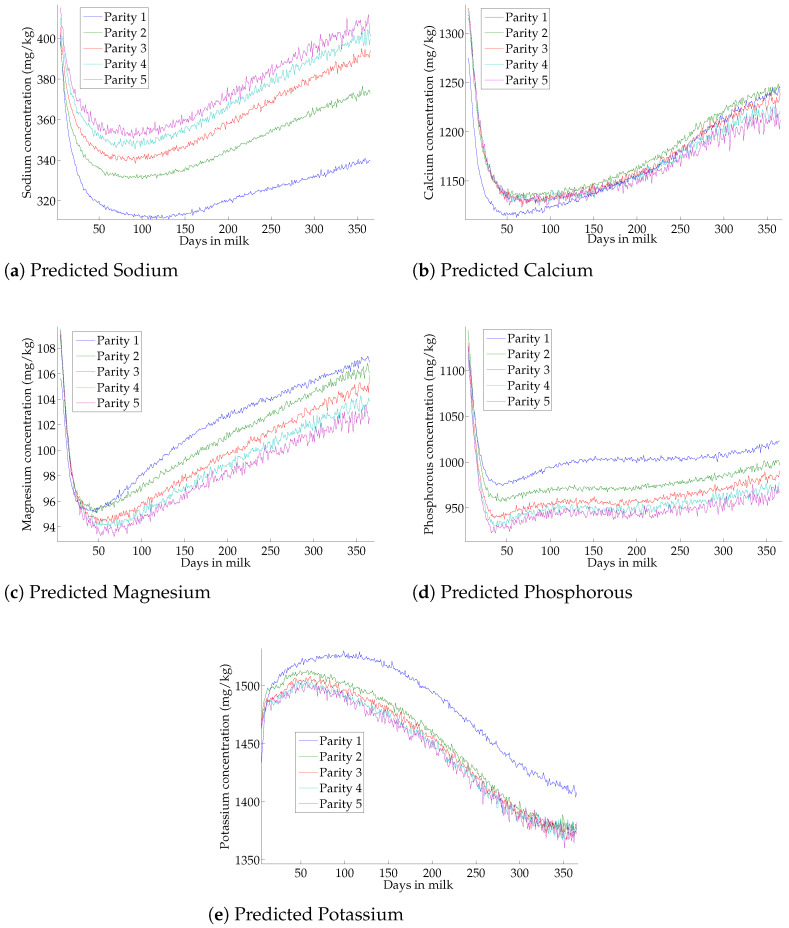
Evolution of the predicted mineral concentration through the days in milk and parity. (**a**) Predicted Sodium (**b**) Predicted Calcium (**c**) Predicted Magnesium (**d**) Predicted Phosphorous (**e**) Predicted Potassium.

The mineral content of milk according to the DIM and the parity is shown in Figure 2. Two behaviors were observed, where the first corresponds to an exponential decrease until the lactation peaks, followed by a slight increase in mineral content over the days for pNa, pCa, pMg, and pP. This trend showed the opposite behavior of milk production. The second behavior corresponds to a slight increase in K content up to the maximum (different according to parity), followed by a slight decrease over the DIM. In agreement with Table 4, potassium was positively correlated with milk yields (r = 0.32). A decrease through the parities was observed for mineral elements (except for pNa and pCa) with a larger gap between the first and second parity.

Analyses of DIM and parity has already been done by different authors [53,54], but these studies focused on the breed and not on a global aspect. Our study has provided a new observation of the mineral concentration depending on the DIM and the parity around 10,000 spectra per days in milk on the first five parities of the cattle. The sharp decrease in mineral traits (except pK) at the beginning of DIM was probably the result of a dilution effect, where the onset of milk yields was indeed very low. The increase of minerals, except for pK, throughout lactation could also be derived from a dilution effect in milk. Moreover, Holt et al. [55] mentioned a rise in casein level in late lactation, leading to a higher content of Ca and Mg, and Kume and Tanabe [56] and Visentin et al. [53] mentioned a similar degressive trend for pCa, pMg, and pP content across parity. Or as in the first parity of cows, the skeleton may not be completely structured, and calcium could be more mobilized for bone fixation instead of calf feeding, explaining the minimum at the beginning and the progressive increase until it is higher than the Ca present in the milk for multiparous cows through the DIM.

A link between mastitis infection and sodium was clearly identified based on the estimated correlations with mastitis indicators (Table 4). The increase in sodium across parity could be explained by the fact that the more cows age, the SCC tends to increase, and the more likely they are to have an udder infection [57,58]. Finally, there was an increase in sodium across lactation, as the increase in SCC is due to the innate immune response of the animal in preparation for calving, and to reinforce the defense mechanism of the mammary gland at this critical period of calving [59] so the sodium would follow the trend of SCC. The correlation between minerals and NAGase could be a good indicator to detect mastitides in dairy cattle.

## 4. Conclusions

In conclusion, this study developed new predictive equations for Na, Ca, Mg, P, and K in milk from a large dataset, including records coming from multiple countries. The robustness of these equations was also demonstrated by external country validations in Austrian samples. The importance of including local variability in the model was tested by integrating 30 Austrian samples in the calibration dataset and validating with the remaining samples. Despite relatively good RMSEP for the model without the addition of the Austrian samples, RMSEP tends to decrease with the addition of the Austrian samples. This work shows the usefulness of including local variability to improve the robustness of a prediction equation.

The correlation values exhibited strong links between Ca, Mg, and P and confirmed the interactions between these minerals and the protein content in milk. The link between pNa and mastitis infection was also confirmed based on the estimated correlations.

Large-scale phenotyping allowed to observe the averaged trends of predicted minerals through the year, the parity, and the days in milk. The concentration of the predicted minerals throughout the year were mainly dependent on the feeding. The predicted mineral concentration tended to decrease through the parity (except for pNa) and increase within the lactation showing an inverse behavior of the milk yields (except for K).

Finally, the potential of mineral profile could be used as a biomarker at an individual level to monitor udder health or technological properties and potentially improve through diet or genetics.

## Figures and Tables

**Table 1:** Equations predicting traits related to animal health and milk technological properties.

Traits	Related Parameter	N Records	Country	RPDcv	Author
Casein (g/100 g)	Cheese process	996	1	4.46	Unpublished
Citrate (mmol/L)	Energy balanced	566	4	3.21	Grelet et al. (2016) [32]
NAGase (Units/L)	Mastitis	377	6	1.41	Unpublished
LDH (Units/L)	Mastitis	305	6	1.21	Unpublished
Lactoferrin (mg/L)	Mastitis	2654	4	1.43	Soyeurt et al. (2020) [34]
RCT (s)	Cheese process	337	1	1.54	Colinet et al. (2015) [35]
k20 (s)	Cheese process	337	1	1.24	Colinet et al. (2015) [35]
a30 (s)	Cheese process	337	1	1.54	Colinet et al. (2015) [35]
Fresh ILCY (g curd/100 g)	Cheese process	337	1	1.91	Colinet et al. (2015) [35]

NAGase: *N*-acetyl-β-d-glucosaminidase; LDH: lactose dehydrogenase RCT: rennet coagulation time; k20: curd-firming time; a30: curd firmness 30 min after rennet addition; ILCY: Individual laboratory cheese yield.

**Table 2:** Descriptive statistics value calculated from reference content (mg/kg of milk) analyzed in ICP-AES.

Mineral	Nsamples	Mean	SD	CV	Min	Max
Na	1145	354.73	90.09	25.40	234.00	1273.00
Ca	1251	1148.40	132.96	11.58	593.40	1743.00
Mg	1281	99.38	13.04	13.12	60.82	156.60
P	1238	990.91	126.31	12.75	508.52	1447.00
K	1246	1513.83	148.88	9.83	819.15	1984.60

**Table 3:** Prediction performances of equations allowing to predict the content (mg/kg of milk) of major minerals in bovine milk.

						10-Fold Cross-Validation				Validation			
Subset	Mineral	Nsamples	Noutliers	LV	$\Delta_{\mathrm{outlier}}$	RMSEcv	R²cv	RPDcv	CCC	RMSEP	R²p	RPDp	CCCp
	Na	1019	26	13	6.41	50.98	0.44	1.34	0.63	55.19	0.36	1.24	0.58
Without	Ca	1094	57	10	46.22	53.38	0.82	2.34	0.90	71.74	0.78	1.74	0.81
Austrian	Mg	1124	57	11	4.28	6.53	0.72	1.88	0.85	8.11	0.51	1.51	0.68
samples	P	1083	55	10	22.72	58.71	0.75	1.99	0.86	81.31	0.83	1.44	0.75
	K	1090	56	12	9.23	88.14	0.55	1.48	0.74	181.48	0.24	0.72	0.20
With 30	Na	997	78	13	7.65	49.05	0.43	1.16	0.58	54.56	0.33	1.04	0.54
Austrian	Ca	1106	75	10	33.04	53.52	0.81	2.31	0.90	63.63	0.77	1.94	0.84
samples	Mg	1164	47	11	13.81	6.87	0.69	1.79	0.83	7.30	0.55	1.69	0.72
included	P	1126	42	10	27.44	62.70	0.72	1.88	0.85	59.47	0.84	2.00	0.84
	K	1120	56	12	8.35	91.30	0.54	1.46	0.73	152.89	0.26	0.87	0.29
80% of	Na	886	31	13	17.73	54.39	0.39	1.15	0.58	62.24	0.49	1.00	0.58
randomly	Ca	961	41	10	29.18	58.30	0.80	2.20	0.89	69.98	0.67	1.83	0.80
selected	Mg	998	27	11	8.51	7.18	0.66	1.70	0.81	7.78	0.69	1.57	0.79
samples	P	965	30	10	6.49	65.04	0.71	1.85	0.84	66.97	0.72	1.79	0.84
	K	965	33	12	14.27	100.17	0.48	1.36	0.74	106.69	0.52	1.28	0.66

Nsample = the numbers of samples included in the models, Noutliers = number of discarded sample after the residual analysis, RMSEcv and RMSEP Standard error of cross-validation and validation respectively; CCC = concordance correlation coefficient, RPD = ratio of prediction to deviation, $\Delta_{outlier}$ (%) model performance increasing with T test (SD = 2.5) and none with the same LV.

**Table 4:** Observed correlations between measured and predicted mineral levels, milk yields ${}^{1}$ (L), and predicted traits as pFat ${}^{2}$ (g/100 g), pProteins ${}^{2}$ (g/100 g), pCasein (g/100 g), pCitrate (mmol/L), pLactose ${}^{2}$ (g/100 g), pNAGase (Units/L), pLDH (Units/L), pLactoferrin (mg/L) pRCT (s), pk20 (s), pa30 (s), and pFresh ILCY (g curd/100 g).

	Reference Minerals					Predicted Minerals				
Traits	Na	Ca	Mg	P	K	pNa	pCa	pMg	pP	pK
pNa	0.68	0.08	0.12	0.04 *	−0.15		0.02	0.06	−0.16	−0.27
pCa	0.00 *	0.86	0.58	0.42	−0.28	0.06 *		0.62	0.51	−0.36
pMg	0.06 *	0.61	0.81	0.53	0.03 *	0.16 *	0.74		0.62	−0.15
pP	0.00 *	0.44	0.53	0.82	−0.18	0.03 *	0.54	0.66		0.13
pK	−0.08	−0.29	−0.16	0.03 *	0.67	−0.14	−0.40	−0.24	0.03 *	
Milk Yields	−0.13	−0.19	−0.22	−0.10	0.28	−0.15	−0.21	−0.27	−0.12	0.32
pFat	−0.04 *	0.27	0.27	0.11	−0.21	−0.09	0.39	0.34	0.11	−0.41
pProteins	0.14	0.58	0.56	0.50	−0.30	0.26	0.68	0.72	0.63	−0.40
pLactose	−0.41	0.07 *	−0.08 *	0.07	−0.06 *	−0.69	0.073	−0.09	0.10	−0.11
pCasein	0.09 *	0.55	0.56	0.51	−0.24	0.17	0.66	0.71	0.64	−0.34
pCitrate	−0.04 *	0.43	0.48	0.12	−0.06 *	−0.02 *	0.54	0.59	0.19	−0.07 *
pNAGase	0.46	0.19	0.32	0.03 *	−0.15	0.74	0.21	0.42	0.06 *	−0.15
pLDH	0.32	0.11	0.24	0.08 *	−0.12	0.54	0.15	0.34	0.00 *	−0.15
pLactoferrin	0.39	0.17	0.08 *	0.03 *	−0.25	0.62	0.18	0.13	0.04	−0.29
pRCT	0.21	0.01 *	−0.01 *	−0.01 *	0.07 *	0.323	−0.06 *	−0.07 *	−0.02 *	0.09 *
pk20	0.06 *	−0.21	−0.25	−0.37	0.09 *	0.098	−0.30	−0.46	−0.28	0.15
pa30	0.04 *	0.23	0.25	0.35	−0.10	0.07 *	0.29	0.45	0.29	−0.14
pFresh ILCY	−0.02 *	0.49	0.49	0.38	−0.25	−0.02 *	0.61	0.58	0.41	−0.43

${}^{1}$ Real milk yields; ${}^{2}$ Manufacturer equation Delta Perkin Elmer; * Correlations between traits who are not significant (*p* > 0.05). Correlations above the diagonal between predicted minerals were performed on the large-scale prediction dataset (real population), and correlations below the diagonal between predicted minerals were performed on the calibration dataset; NAGase: *N*-acetyl-β-d-glucosaminidas; LDH: lactose dehydrogenase; RCT: rennet coagulation time; k20: curd-firming time; a30: curd firmness 30 min after rennet addition; ILCY: Individual laboratory cheese yield.

**Table 5:** Descriptive statistics of large-scale phenotyping on the mid-infrared predictions Walloon database. The means, standard deviation, and minimum and maximum values are expressed in mg/kg.

Mineral	N	Mean	SD	Min	Max
Na	3,510,077	346	43	200	500
Ca	3,510,077	1163	102	900	1500
Mg	3,510,077	100	8	80	130
P	3,510,077	976	91	700	1300
K	3,510,077	1471	98	1100	1700

## Abbreviations

The following abbreviations are used in this manuscript:

DHI	Dairy Herd Improvement
ICP-AES	Inductively Coupled Plasma—Atomic Emission Spectroscopy
GH	Malabonis distance
NH	Neighboring H distance
RMSE	Root Mean Square Error
MIR	Mid-infrared
PDS	Piece-wise standardized method
PLS	Partial least square regression
RMSEcv	Root Mean Square Error of cross-validation
RMSEP	Root Mean Square Error of prediction
RPD	Ratio of prediction to deviation
DIM	Days in milk
pX	Predicted traits (X = Na, Ca, Mg, P, K, etc.)
NAGase	*N*-acetyl-β-D-glucosaminidase
LDH	Lactase Lactate dehydrogenase
RCT	Rennet coagulation time
k20	Curd-firming time
a30	Curd firmness 30 min after rennet addition
ILCY	Individual laboratory cheese yield
